# Levosimendan Improves Oxidative Balance in Cardiogenic Shock/Low Cardiac Output Patients

**DOI:** 10.3390/jcm9020373

**Published:** 2020-01-30

**Authors:** Elena Grossini, Serena Farruggio, Daniele Pierelli, Virginia Bolzani, Lidia Rossi, Piero Pollesello, Carolina Monaco

**Affiliations:** 1 Laboratory of Physiology, Department of Translational Medicine, UPO, 28100 Novara, Italy; serefar@live.it; 2 Cardiothoracic Intensive Care Unit, AOU, 28100 Novara, Italy; daniele.pierelli@gmail.com (D.P.); carolina.monaco@maggioreosp.novara.it (C.M.); 3 Cardiology Division, AOU, 28100 Novara, Italy; virgi.bolzani@gmail.com (V.B.); rossilidia7@gmail.com (L.R.); 4 Critical Care, OrionPharma, 02101 Espoo, Finland; piero.pollesello@orionpharma.com

**Keywords:** antioxidants, calcium sensitizer, heart failure, mitochondria function, peroxidation

## Abstract

The beneficial effects exerted by levosimendan against cardiac failure could be related to the modulation of oxidative balance. We aimed to examine the effects of levosimendan in patients with cardiogenic shock or low cardiac output on cardiac systo-diastolic function and plasma oxidants/antioxidants (glutathione, GSH; thiobarbituric acid reactive substances, TBARS). In four patients undergoing coronary artery bypass grafting or angioplasty, cardiovascular parameters and plasma GSH and TBARS were measured at T0 (before levosimendan infusion), T1 (1 h after the achievement of the therapeutic dosage of levosimendan), T2 (end of levosimendan infusion), T3 (72 h after the end of levosimendan infusion), and T4 (end of cardiogenic shock). We found an improvement in the indices of systolic (ejection fraction, cardiac output, cardiac index) and diastolic (E to early diastolic mitral annular tissue velocity, E/’; early to late diastolic transmitral flow velocity, EA) cardiac function at early T2. A reduction of central venous pressure and pulmonary wedge pressure was also observed. Plasma levels of GSH and TBARS were restored by levosimendan at T1, as well. The results obtained indicate that levosimendan administration can regulate oxidant/antioxidant balance as an early effect in cardiogenic shock/low cardiac output patients. Modulation of oxidative status on a mitochondrial level could thus play a role in exerting the cardio-protection exerted by levosimendan in these patients.

## 1. Introduction

Levosimendan is an inotrope used for the treatment of acutely decompensated heart failure patients with low cardiac output or cardiogenic shock [1,2,3,4].

Among its mechanisms of action, the sensitization of cardiac troponin C to calcium in cardiac muscle [5,6,7] and the opening adenosine triphosphate-sensitive potassium (K_ATP_) channels in vascular smooth muscle cells [8,9] have been described. Due to these pharmacological actions, levosimendan improves atrio-ventricular coupling and cardiac mechanical efficiency without increasing myocardial oxygen consumption [10,11,12,13]. Moreover, levosimendan has been shown to have a direct effect on mitochondria [14,15,16,17,18,19].

Preliminary data obtained in vitro, ex vivo, and in vivo in animal models have shown that levosimendan could improve endothelial and mitochondrial function, and protect against peroxidation, as well. In anesthetized pigs subjected to renal ischemia/reperfusion, the intrarenal levosimendan administration was able to increase renal function and keep the oxidant balance, as shown by the increased plasma levels of glutathione (GSH) and the reduced release of peroxidation markers, like thiobarbituric acid reactive substances (TBARS) [20]. Similar nominally beneficial effects against peroxidation were observed in anesthetized rats subjected to hepatic ischemia/reperfusion and treated with levosimendan [21].

TBARS, which reflect the release of malon(MDA) generated through the peroxidation of polyunsaturated fatty acids, have been widely adopted as indicators of oxidative stress in various cardiovascular diseases [22]. GSH plays an important role in the maintenance of the thiol-redox status of the cell, and for this reason could act as antioxidant. GSH deficiency might manifest itself through an increased susceptibility to oxidative stress and to the augmented onset and progression of many diseases, including the cardiovascular diseases [23].)

Similar beneficial effects against peroxidation, as were observed in the renal model of ischemia/reperfusion, have been observed in anesthetized rats subjected to hepatic ischemia/reperfusion and treated with levosimendan [21]. Finally, in rat hepatocytes, the administration of levosimendan dose-dependently counteracted the injuries caused by oxidative stress, as evidenced by the keeping GSH content and the reduction of TBARS release [24]. In all the above conditions, endothelial and mitochondrial function were found to be ameliorated by levosimendan, which prevented the fall of mitochondrial membrane potential and restored nitric oxide (NO) release.

The beneficial effects elicited by levosimendan against oxidative stress could represent a further mechanism of protection in heart failure (HF) patients. The implication of oxidative stress in the pathophysiology of HF is well established. Oxidative stress may impair cardiac functions through damage to the cellular proteins and membranes by initiation of lipid peroxidation, thereby inducing cellular death and apoptosis. Moreover, it could exert direct negative inotropic effects through the reduction of cytosolic intracellular free calcium [25]. Thus, there is strong circumstantial evidence that oxidative stress is a prognostic factor in HF patients. Keith et al. showed that circulating MDA, a marker of lipid peroxidation, was significantly different between control subjects and patients with HF [26]. In addition, MDA plasma levels have been positively correlated not only with the presence of HF, but also with New York Heart Association (NYHA) functional class [27]**.**

It is worth noting that levosimendan administration to decompensated HF subjects was able to improve oxidative damage, as shown by the TBARS measurement, by five days [28]. To date, however, the information available about the effects of levosimendan on oxidative stress and cardiac systo-diastolic function in cardiogenic shock/low cardiac output patients for ischemic disease, is scarce.

For this reason, in the present study, we aimed to examine, at the same time, the effects of intravenous levosimendan treatment on (1) systolic and diastolic functions, (2) hemodynamic variables, and (3) oxidant/antioxidant systems, in patients admitted to the cardiothoracic intensive care unit (ICU) for cardiogenic shock or decompensated heart failure after coronary artery bypass grafting (CABG) or percutaneous transluminal coronary angioplasty (PTCA). To our knowledge, this is the first time these parallel assessments have been performed in a clinical setting.

## 2. Materials and Methods

This was a prospective, longitudinal study approved by the Ethical Committee of Azienda Ospedaliero Universitaria (AOU) Maggiore della Carità of Novara (655/CE; Studio n. CE 107/17; approval date 16/06/2017). All procedures were compliant with the ethical standards of the Helsinki Declaration and conformed to standards currently applied in Italy. Patients’ informed written consent was collected before starting the study.

In this pilot study, 4 adult patients admitted to the cardiothoracic ICU of AOU Maggiore della Carità of Novara were enrolled.

Inclusion criteria were: Aged over 18 years, reduced systolic function (ejection fraction, EF, < 30%) due to cardiogenic shock post-CABG or decompensated heart failure after PTCA.

Exclusion criteria: Acute renal or liver failure, septic condition.

Two male patients underwent CABG for three vessel-diseases. In PTCA patients (one male and one female), three arterial grafts were positioned on the left anterior descending coronary artery, and intra-aortic balloon pumps (IABP) were used. Demographic data are reported in Table 1.

Hemodynamic variables were monitored by means of a Swan–Ganz pulmonary artery catheter in all patients [29,30].

The central venous pressure (CVP), mean pulmonary arterial pressure (PAP), cardiac output (CO), cardiac index (CI), systemic vascular resistance index (SVRI), pulmonary vascular resistance index (PVRI), and the pulmonary capillary wedge pressure (PCWP) were continuously measured. Heart rate (HR), and systolic (SAP) and diastolic (DAP) arterial blood pressure were also recorded. Arterial and venous oxygen partial pressure (pO_2_), and arterial and venous oxygen saturation (SO_2_) were examined by hemogasanalyis (Radiometer ABL 90 Flex).

In all patients, systo-diastolic cardiac function was examined by an expert cardiologist through echocardiography (GE Vivid-i), following the time-course reported below, and as recommended by international guidelines [31,32,33].

After baseline data evaluation, levosimendan was administered intravenously at the dose of 0.1 μg/kg/min without a loading dose, which is adopted in the ICU for the treatment of cardiogenic shock or low cardiac output patients [34,35]. In all patients, vasopressor support was implemented by epinephrine (0.01–0.1 μg/kg/min) and in one patient with dopamine (2 μg/kg/min), as well. Mechanically assisted ventilation was provided to CABG patients.

Venous samples were taken for GSH and TBARS measurements, following the time-course reported below.

### 2.1. Collection of Samples

For the determination of GSH and TBARS, 10 mL of blood samples were taken from each donor using BD Vacutainer tubes (sodium heparin as anticoagulant). Each sample was immediately centrifuged by a refrigerated centrifuge (Eppendorf, mod. 5702 with rotor A-4-38) for 10 min, at a speed of 3100 g at 4 °C. The plasma obtained was divided into 5 tubes that were stored at −20 °C at the Physiology laboratory of the University of Eastern Piedmont of Novara.

### 2.2. GSH Quantification

GSH measurement was performed by using the Glutathione Assay Kit (Cayman Chemical, Ann Arbor, MI, USA), as previously described [36,37]. Each plasma sample was deproteinated by adding an equal volume of MPA solution to the sample which was then centrifuged at 2000 g for 2 min. The supernatant was collected, and 50 μL/mL of TEAM reagent was added to each sample in order to increase the pH. Fifty microliters of the samples were transferred to a 96-well plate where GSH was detected following the manufacturer’s instructions through a spectrophotometer (VICTOR™ X Multilabel Plate Reader), at excitation/emission wavelengths of 405–414 nm. Glutathione was expressed as µM. The measurements were performed in triplicate.

### 2.3. TBARS Quantification

TBARS were determined as MDA release. The MDA measurement was performed by using the TBARS assay Kit (Cayman Chemical), as previously [36,38]. For the assays, 100 µL of each plasma sample was added to 100 µL of sodium dodecyl sulfate (SDS) solution and 2 mL of the Color Reagent, following the manufacturer’s instructions. Each sample was boiled for 1 h and then transferred to ice for 10 min in order to stop the reaction. Each sample was centrifuged for 10 min at 1600 g at 4 °C and 150 μL was transferred to a 96-well plate where MDA was detected following the manufacturer’s instructions through a spectrophotometer (VICTOR™ X Multilabel Plate Reader), at excitation/emission wavelengths of 530–540 nm. MDA production was expressed in µM. The measurements were performed in triplicate.

### 2.4. Time-Course of Measurements

T0, before the beginning of levosimendan administration: Hemodynamic variable measurements and cardiac systo-diastolic evaluation, GSH and TBARS sampling. 

T1, at the end of 0.1 μg/kg/min levosimendan infusion (duration 24 h): Hemodynamic variable measurements and cardiac systo-diastolic evaluation, GSH and TBARS sampling.

T2, 24 h after the end of levosimendan infusion: Hemodynamic variable measurements and cardiac systo-diastolic evaluation, GSH and TBARS sampling.

T3, at 72 h after the end of levosimendan administration: Hemodynamic variable measurements and cardiac systo-diastolic evaluation, GSH and TBARS sampling.

T4, at the end of inotropic support: Hemodynamic variable measurements and cardiac systo-diastolic evaluation, GSH and TBARS sampling.

### 2.5. Statistical Analysis

All data were recorded using the Institution’s database. Statistical analysis was performed by using GraphPad Prism 6.0. Non-nominal variables were checked for normality before statistical analysis. ANOVA for repeated measurements was used to compare results obtained in each patient at various timings. All non-nominal data were expressed as means ± standard deviation (SD). A *p* value lower than 0.05 was taken for statistical significance.

## 3. Results

The patients were overweight, and one patient was diabetic, but major cardiovascular risk factors like hypertension, smoking, or dyslipidemia were not identified (Table 1). At T0, mean EF amounted to 25%. In the two patients who underwent cardiothoracic surgery, extracorporeal circulation lasted 145 ± 21 min. 

**Figure 1:**
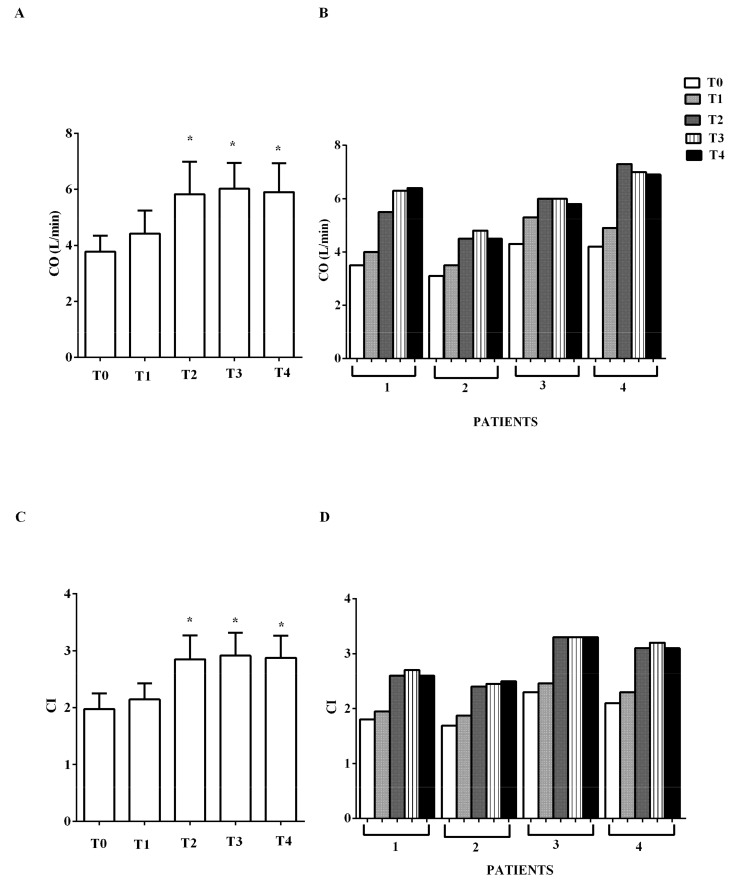
In (**A**,**B**), effects of levosimendan on cardiac output (CO) and in (**C**,**D**), on cardiac index (CI). In A and C, values are means ± SD. In B and D, columns represent single patients. * *p* < 0.05 vs. T0.

**Figure 2:**
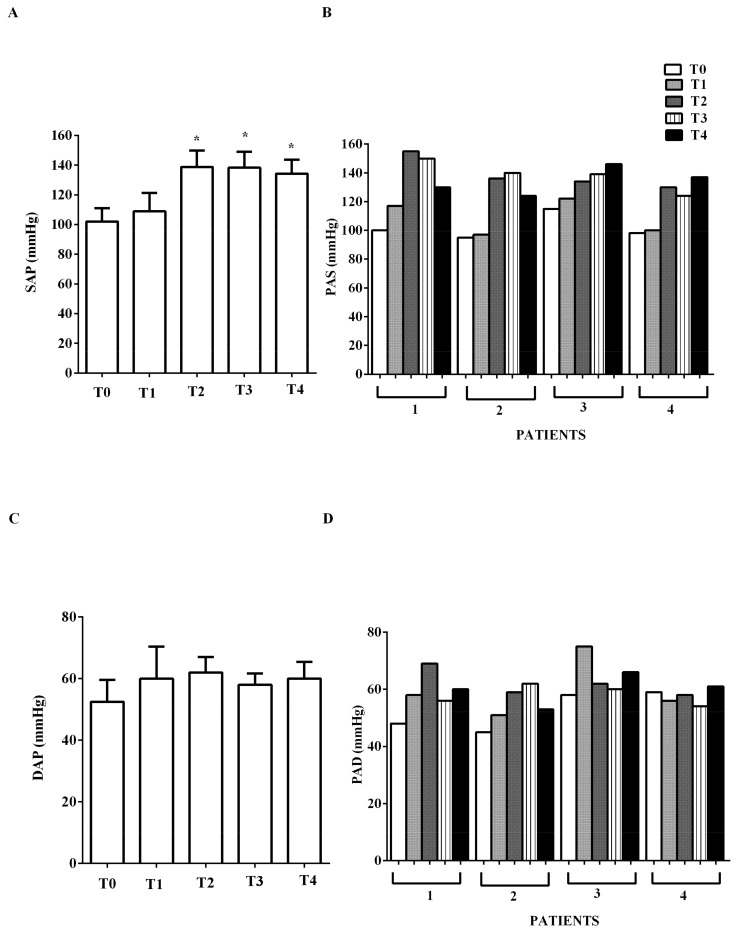
In (**A**,**B**), effects of levosimendan on systolic (SAP) and in (**C**,**D**), diastolic (DAP) arterial blood pressure. In A and C, values are means ± SD. In B and D, columns represent single patients. * *p* < 0.05 vs. T0.

Improvements were recorded in CO, CI, and SAP, as shown in Figure 1 and Figure 2A,B.

**Figure 3:**
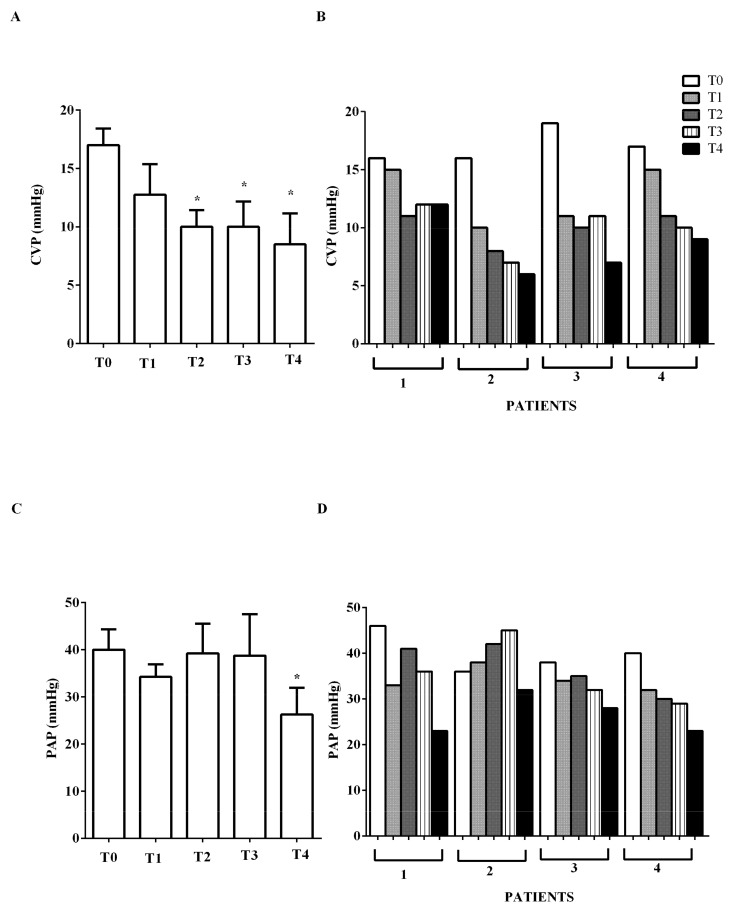
In (**A**,**B**), effects of levosimendan on central venous pressure (CVP) and in (**C**,**D**) on mean pulmonary arterial pressure (PAP). In A and C, values are means ± SD. In B and D, columns represent single patients. * *p* < 0.05 vs. T0.

**Figure 4:**
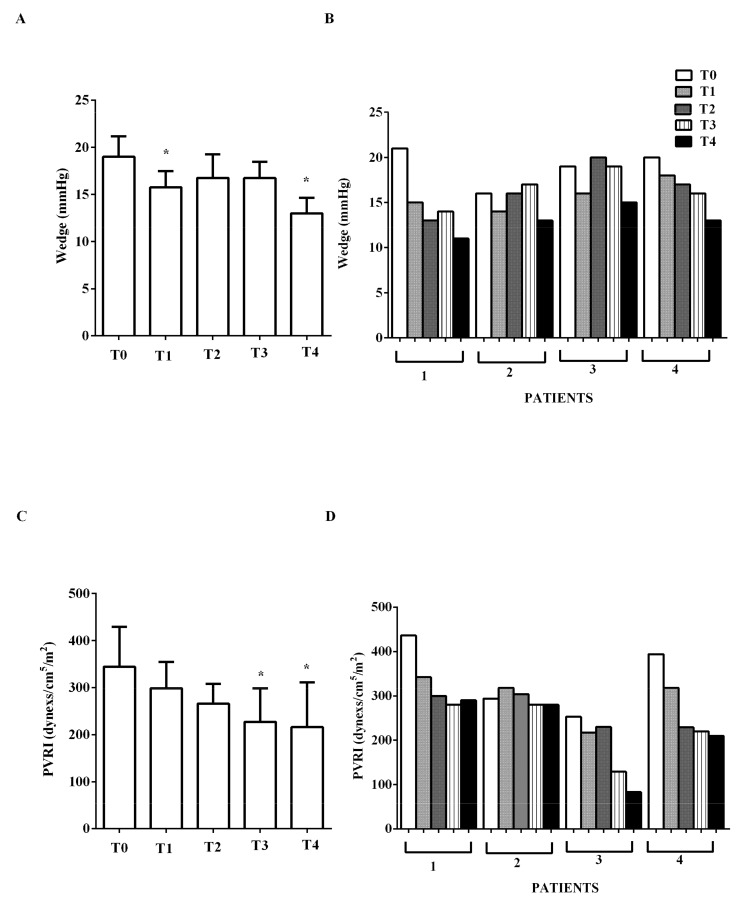
In (**A**,**B**), effects of levosimendan on pulmonary capillary wedge pressure (wedge) and in (**C**,**D**), on pulmonary vascular resistance index (PVRI). In A and C, values are means ± SD. In B and D, columns represent single patients. * *p* < 0.05 vs. T0.

EF increased from mean 26.25% ± 2.2 to 43.7% ± 2.9 at early T2, and to 48% ± 1.4 at T4 (*p* < 0.05). A reduction of CVP, pulmonary capillary wedge pressure (wedge), and PVRI was also observed (Figure 3A,B and Figure 4B,D). PAP at T4 was lower than PAP at T0 (Figure 3C,D). 

Indices of diastolic function (E/E’, E/A) were improved by levosimendan administration (E to early diastolic mitral annular tissue velocity, E/, from mean 14.5 ± 1.3 at T0, to mean 11 ± 1.4 at T2, to mean 6.7 ± 1.7 at T4; early to late diastolic transmitral flow velocity, E/A, from >1 at T0 to <1 at T4; *p* < 0.05). No significant changes of HR were observed (mean values at T0 to T4, respectively: 96.75, 90.75, 95.75, 88, 91 beats/min), nor in DAP (Figure 2C,D). In the two PTCA patients, IABP was removed at T2 and mechanical ventilation was suspended after 1 and 3 days, respectively. In all patients, epinephrine was reduced from mean 0.06 ± 0.04 μg/kg/min at T0, to 0.04 ± 0.02 μg/kg/min at T2, to 0.001 ± 0.009 μg/kg/min at T3; at T4 it was suspended. Dopamine was reduced from 2 μg/kg/min at T0, to 1 μg/kg/min at T2; at T4 it was suspended.

**Figure 5:**
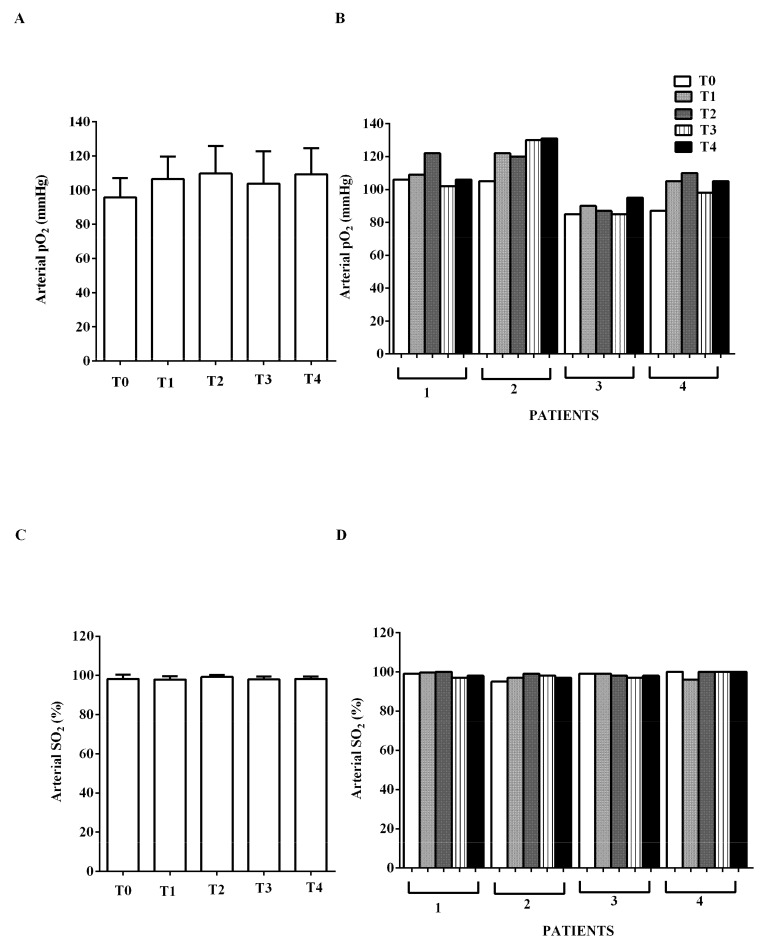
In (**A**,**B**), effects of levosimendan on arterial partial pressure (pO_2_) and in (**C**,**D**), on arterial oxygen saturation (SO_2_). In A and C, values are means ± SD. In B and D, columns represent single patients.

**Figure 6:**
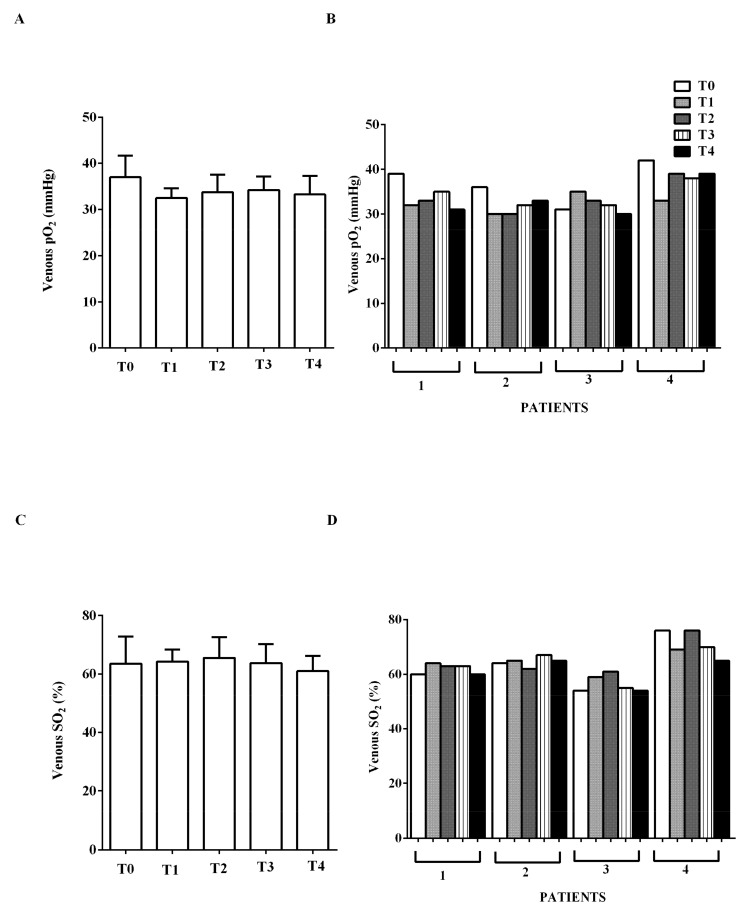
In (**A**,**B**), effects of levosimendan on venous oxygen partial pressure (pO_2_) and in (**C**,**D**), on venous oxygen saturation (SO_2_). In A and C, values are means ± SD. In B and D, columns represent single patients.

Arterial oxygen saturation and oxygen partial pressure amounted to about 98% and 96 mmHg at T0 and did not vary significantly throughout the time-course (Figure 5).

ygen venous partial pressure and venous oxygen saturation amounted to about 37 mmHg and 63% at T0 and did not show any changes (Figure 6).

**Figure 7:**
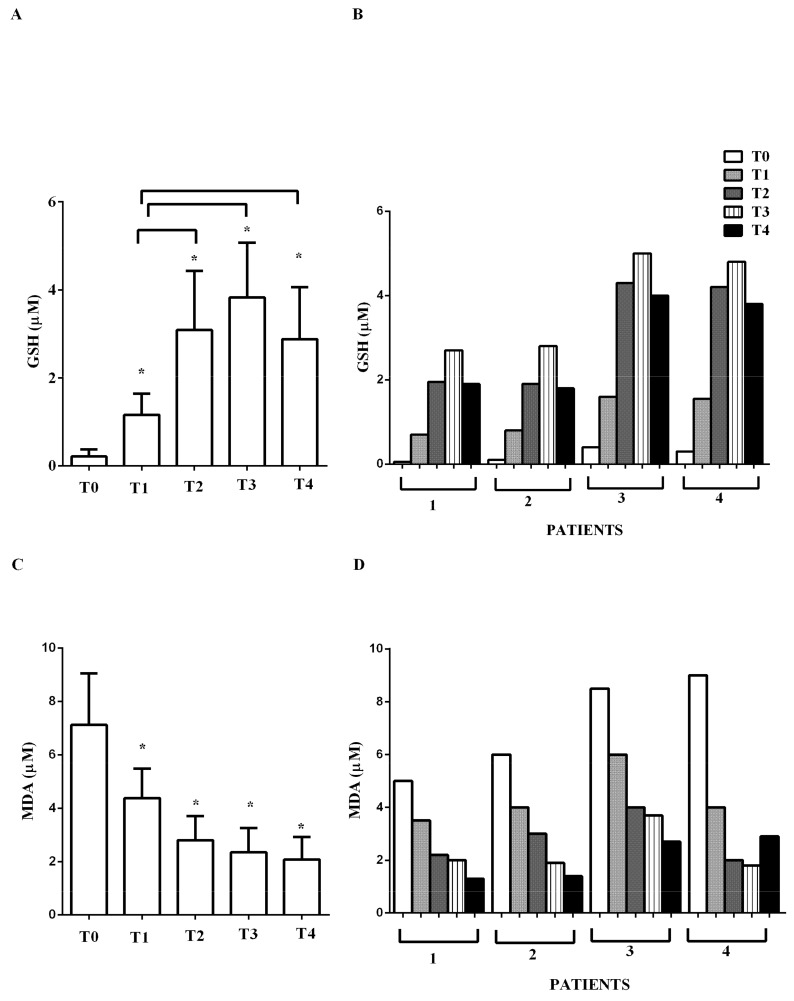
In (**A**,**B**), effects of levosimendan on plasma glutathione (GSH) and in (**C**,**D**), on plasma malonyldialdeide (MDA) concentration. In A and C, values are means ± SD. In B and D, columns represent single patients. * *p* < 0.05 vs. T0.

As depicted in Figure 7, at T0, the levels of GSH and MDA were 0.21 ± 0.16 μM and 7.1 ± 1.9 μM, respectively. Immediately after levosimendan administration, an improvement of the above parameters was observed. GSH at T1 rose to 1.16 ± 0.5 μM, whereas MDA declined to 4.3 ± 1.1 μM (both *p* < 0.05 vs. T0). At T2, T3, and T4, the levels of GSH increased (3.1 ± 1.3; 3.8 ± 1.2; 2.8 ± 1.2 μM, respectively; all *p* < 0.05 vs. T0) and those of MDA decreased progressively (2.5 ± 0.9; 2.4 ± 0.9; 2.1 ± 0.8 μM; all *p* < 0.05 vs. T0).

## 4. Discussion

The results obtained in the present study show protective effects elicited by levosimendan administration in cardiogenic shock or low cardiac output patients post-CABG or PTCA, through the modulation of oxidant/antioxidant balance.

The balance between ROS production and their removal by antioxidant systems is described as the “redox state”. Oxidative stress arises any time the production of ROS exceeds the levels of antioxidants. In HF, an increased production of ROS, originating mainly by mitochondria from failing hearts, has been widely evidenced [39]. Chronic increases in ROS production may lead to a cycle of mitochondrial DNA damage, leading to a functional decline, further ROS generation, and cellular injury. ROS can directly impair contractile function by modifying proteins central to excitation–contraction coupling, ion transporters, and Ca^2+^ cycling, as well [40].

It is noteworthy that cardiac oxidative stress has been associated with diastolic dysfunction, as well [41], via changes in Ca^2+^ handling. Hence, increased ROS production can impair the activity of Ca^2+^/calmodulin kinase (CaMK) II or SERCA2, or affect Ca^2+^ sensitivity of myofilaments. As a result, a diastolic SR Ca^2+^ leak and a reduction of relaxation stiffness of cardiomyocytes would happen [42].

In addition, changes of mitochondrial function could lead to increased intracellular Ca^2+,^ resulting in cardiomyocyte super-contracture, disruption of plasmalemma and therefore necrotic cell death. Although it was not clearly shown, at the basis of the above effects there could be the increased mitochondrial ROS generation [43].

Furthermore, KA_TP_ channels could represent a target for ROS in HF [44]. It has been shown that ROS-dependent modification of mitochondrial K_ATP_ channels, in particular, could represent a feedback mechanism for the regulation of mitochondrial K_ATP_ channel activity itself. Moreover, K_ATP_ channels have been widely shown to play important roles in protection of the heart from ischemic injury [45]. The opening of K_ATP_ channels could contribute to the regulation of cardiac mitochondrial function [46] and cardioprotection induced by ischemic preconditioning, as well.

For this reason, any factor able to affect K_ATP_ channel activity could represent a rescue method against cardiac damage.

The mechanisms of action of levosimendan are mainly related to the increase of Ca^2+^ sensitivity of troponin C and the opening of K_ATP_ channels in myocardium and vessels. Being a calcium sensitizer and not a calcium mobilizer, levosimendan does not increase myocardial oxygen consumption nor prevent myocardial apoptosis and remodeling [1].

For those reasons, levosimendan is widely used for the treatment of low cardiac output conditions for its effects on systemic and pulmonary hemodynamics and for the relief of symptoms of HF [47]**.**

Recent evidence indicates that levosimendan could reduce oxidative markers and increase the antioxidant system. In decompensated HF patients, a reduction of TBARS levels was observed after 5 days from the start of levosimendan administration [28], while, in the animal model of renal and liver ischemia/reperfusion plasma, TBARS and GSH concentration was restored by levosimendan infusion [20,21]. A role for the modulation of the “redox state”, as a possible mechanism of action of levosimendan in the protection against HF, might therefore be hypothesized.

In our study, the infusion of levosimendan in cardiogenic shock or low cardiac output patients post-CABG or PTCA improved GSH and reduced TBARS early at the achievement of the therapeutic levosimendan dosage. Those effects preceded those on hemodynamics and cardiac systo-diastolic function, which were evidenced by the improvement of EF, CO, CI, E/E’, and E/A.

Our data confirm previous data about the protective effects exerted by levosimendan against peroxidation, and highlight their potential as further mechanisms through which levosimendan could exert its action on cardiac function. Although it was not examined, the maintenance of mitochondria function by levosimendan could be presumed to play a role in its antioxidant effects.

Hence, mitochondria have emerged as a central factor in the pathogenesis and progression of HF. It is widely accepted that mitochondrial dysfunction can contribute to impaired myocardial energetics and increased oxidative stress in cardiomyopathies, cardiac ischemic damage, and HF. Mitochondrial permeability transition pore opening has been shown to act as a critical trigger of myocyte death and myocardial remodeling. Increased mitochondrial permeabilization is a mechanistic pathway at the basis of myocardial apoptosis [48].

Previous findings have evidenced that levosimendan is able to prevent the fall of mitochondrial membrane potential and transition pore opening in cardiomyocytes [49]. In this way, it could modulate ROS release and apoptotic signaling. Thus, it could be hypothesized that the keeping mitochondrial function by levosimendan could represent the starting mechanism for the reduction of ROS release and the restoration of myocardial energetics and cell viability. These events would be followed by the improvement of cardiac systole and diastole and hemodynamics.

In conclusion, the results obtained have shown that levosimendan improved GSH and reduced TBARS early before the onset of the cardiovascular effects.

The main limitation of this study is the low number of patients and the different etiologies. However, the aim of this pilot study was to evaluate if levosimendan could exert its protective effects in patients with cardiac low output syndrome or cardiogenic shock on cardiovascular function and hemodynamics as a result of its ability to prevent the GSH reduction and TBARS increase, regardless of the etiology. Moreover, no comparison was performed with a “control” group. However, in this study, all patients were in control of themselves and we analyzed, at different time points, the concomitant changes of hemodynamic variables and cardiac systo-diastolic function, as well as the changes in the levels of oxidants and antioxidants. The results obtained have shown that levosimendan improved GSH and reduced TBARS early before the onset of the cardiovascular effects. Thus, in spite of the low number of cases, levosimendan infusion was able to significantly exert beneficial effects on both the oxidant and antioxidant system, hemodynamics, and cardiac function in cardiogenic shock/low cardiac output patients. A larger number of cases will be necessary to perform a more detailed analysis of the protective effects of levosimendan and evaluation of any differences among various patients.

Overall, the results obtained in this pilot study set the rationale for a clinical trial in a larger population, whose number will be established through a power calculation, and stratified for age, sex, BMI, comorbidities, and etiologies. In this regard, the role of IABP as a modulator of oxidative stress could be further analyzed. In addition, it would be interesting to collect more data about the “redox state” and inflammation and compare the effects of levosimendan with those of other inotropes.

Finally, it seems important to follow the putative anti-inflammatory effects of this drug, not only in cardiac patients, but also in patients with conditions such as pulmonary hypertension or amyotrophic lateral sclerosis, in which levosimendan is currently being clinically evaluated [50,51].

## Figures and Tables

**Table 1:** Demographic data of patients.

Males/Females	3/1
Age (years)	63 ± 13.5
BMI (body mass index)	26.63 ± 1.2
Diabetes	1/4
Hypertension	0
Smoker (>1 cigarette)	0
Dyslipidemia	0
